# Relationship between ion migration and interfacial degradation of CH_3_NH_3_PbI_3_ perovskite solar cells under thermal conditions

**DOI:** 10.1038/s41598-017-00866-6

**Published:** 2017-04-26

**Authors:** Seongtak Kim, Soohyun Bae, Sang-Won Lee, Kyungjin Cho, Kyung Dong Lee, Hyunho Kim, Sungeun Park, Guhan Kwon, Seh-Won Ahn, Heon-Min Lee, Yoonmook Kang, Hae-Seok Lee, Donghwan Kim

**Affiliations:** 1 Department of Materials Science and Engineering, Korea University, 145 Anam-ro, Seongbuk-gu, Seoul, 02841 Republic of Korea; 2 Materials & Production engineering Research Institute, LG Electronics, 38 Baumoe-ro, Seocho-gu, Seoul, 06763 Republic of Korea; 3 KU·KIST Green School Graduate School of Energy and Environment, Korea University, 145 Anam-ro, Seongbuk-gu, Seoul, 02841 Republic of Korea

## Abstract

Organic-inorganic hybrid perovskite solar cells (PSCs) have been extensively studied because of their outstanding performance: a power conversion efficiency exceeding 22% has been achieved. The most commonly used PSCs consist of CH_3_NH_3_PbI_3_ (MAPbI_3_) with a hole-selective contact, such as 2,2′,7,7′-tetrakis(*N*,*N*-di-*p*-methoxyphenylamine)-9,9-spiro-bifluorene (spiro-OMeTAD), for collecting holes. From the perspective of long-term operation of solar cells, the cell performance and constituent layers (MAPbI_3_, spiro-OMeTAD, etc.) may be influenced by external conditions like temperature, light, etc. Herein, we report the effects of temperature on spiro-OMeTAD and the interface between MAPbI_3_ and spiro-OMeTAD in a solar cell. It was confirmed that, at high temperatures (85 °C), I^−^ and CH_3_NH_3_^+^ (MA^+^) diffused into the spiro-OMeTAD layer in the form of CH_3_NH_3_I (MAI). The diffused I^−^ ions prevented oxidation of spiro-OMeTAD, thereby degrading the electrical properties of spiro-OMeTAD. Since ion diffusion can occur during outdoor operation, the structural design of PSCs must be considered to achieve long-term stability.

## Introduction

Since the advent of organic-inorganic hybrid perovskite solar cells (PSCs) in 2009, such cells have been extensively researched. PSCs offer the benefits of a simple and cost-effective fabrication process and outstanding power conversion efficiency^1–5^. Recently, PSCs with >22% efficiency were reported; however, the long-term stability of these types of solar cells impedes their commercialization^6^. High-efficiency PSCs consist of a perovskite absorber, such as MAPbI_3_ or Cs_x_(MA_y_FA_1−y_)_(1−x)_Pb(I_z_Br_1−z_)_3_, with respective electron- and hole-selective contacts such as mesoporous TiO_2_ (m-TiO_2_) and 2,2′,7,7′-tetrakis(*N*,*N*-di-*p*-methoxyphenylamine)-9,9-spiro-bifluorene (spiro-OMeTAD)^5, 7^.

An efficiency drop has frequently been reported for these PSCs. The primary causes of degradation of the performance of solar cells are widely known to be related to external conditions, such as moisture^8, 9^, temperature^10, 11^, UV light^12, 13^, etc. In addition, issues related to internal factors, such as ion migration^14, 15^, interfacial reactions^16^, etc., are stated as causes of insufficient long-term stability of PSCs. Solar cells must exhibit stability to the external conditions stated above to operate efficiently for several decades. Since the temperature of the solar cell can reach up to 85 °C during typical operating conditions, thermal stability is important for long-term operation in particular.

In the m-TiO_2_/MAPbI_3_/spiro-OMeTAD structure, spiro-OMeTAD acts as a hole collector and also protects MAPbI_3_ from the external environment. In recent research, the chemical structure of spiro-OMeTAD was modified to increase its glass transition temperature and conductivity to enhance its thermal stability and hole collection ability^17^. To protect the perovskite from moisture, various hole transporting materials (HTMs), such as hydrophobic and inorganic materials, have been developed resulting in increased long-term stability^18–21^. They enhanced not only the long-term stability of PSCs, but also the electrical contact of perovskite/HTM interfaces. Since spiro-OMeTAD suffers from low electrical conductivity due to the large intermolecular distances^22, 23^, dopants like Li-bis(trifluoromethanesulfonyl) imide (Li-TFSI) and tert-butylpyridine (TBP) have been added to spiro-OMeTAD to induce the doping effect^24^. It has been reported that perovskite materials may be decomposed by the liquids used for deposition of HTMs, such as TBP and acetonitrile^25, 26^; thus, HTM-free PSCs have been fabricated to enhance their long-term stability^27^. However, these kinds of PSCs have lower efficiencies than PSCs with traditional structures including of HTM.

The relationship between MAPbI_3_ and spiro-OMeTAD was evaluated; the results showed that under photo-irradiation with >450-nm wavelength light, MAPbI_3_ assisted the oxidation of spiro-OMeTAD^28^. In contrast, introduction of CH_3_NH_3_I (MAI) into spiro-OMeTAD can reduce the oxidized spiro-OMeTAD (spiro-OMeTAD^+^)^29^. Thus, studies on the interfacial reaction between MAPbI_3_ and spiro-OMeTAD under various external environments are needed to achieve long-term stability.

In this study, the degradation of spiro-OMeTAD and the MAPbI_3_/spiro-OMeTAD interface due to ion migration is investigated with variation of the thermal conditions. While there was no significant change of MAPbI_3_ at 85 °C within a short time, spiro-OMeTAD and the interface between MAPbI_3_/spiro-OMeTAD were affected because of migration of I^−^ and MA^+^. Analysis of the results revealed that PSCs can be thermally degraded due to ion migration, thereby reducing the conductivity and hole collection ability of spiro-OMeTAD.

## Results and Discussions

A methylammonium lead iodide perovskite layer was obtained by spin-coating using a one-step solution process via dripping diethyl ether^7^. In this study, the thermal behavior was evaluated based on reverse scanning of the solar cells with ~16% power conversion efficiency (Figure S1). The external quantum efficiency (EQE) was nearly 80% at wavelengths of 400–600 nm. No PbI_2_ peak (12.6° (001)) was observed in the XRD pattern of the prepared m-TiO_2_/MAPbI_3_ substrate, while typical peaks for MAPbI_3_ were observed at 14.1° (110), 19.9° (112), 23.5° (211), and 24.5° (202).

**Figure 1:**
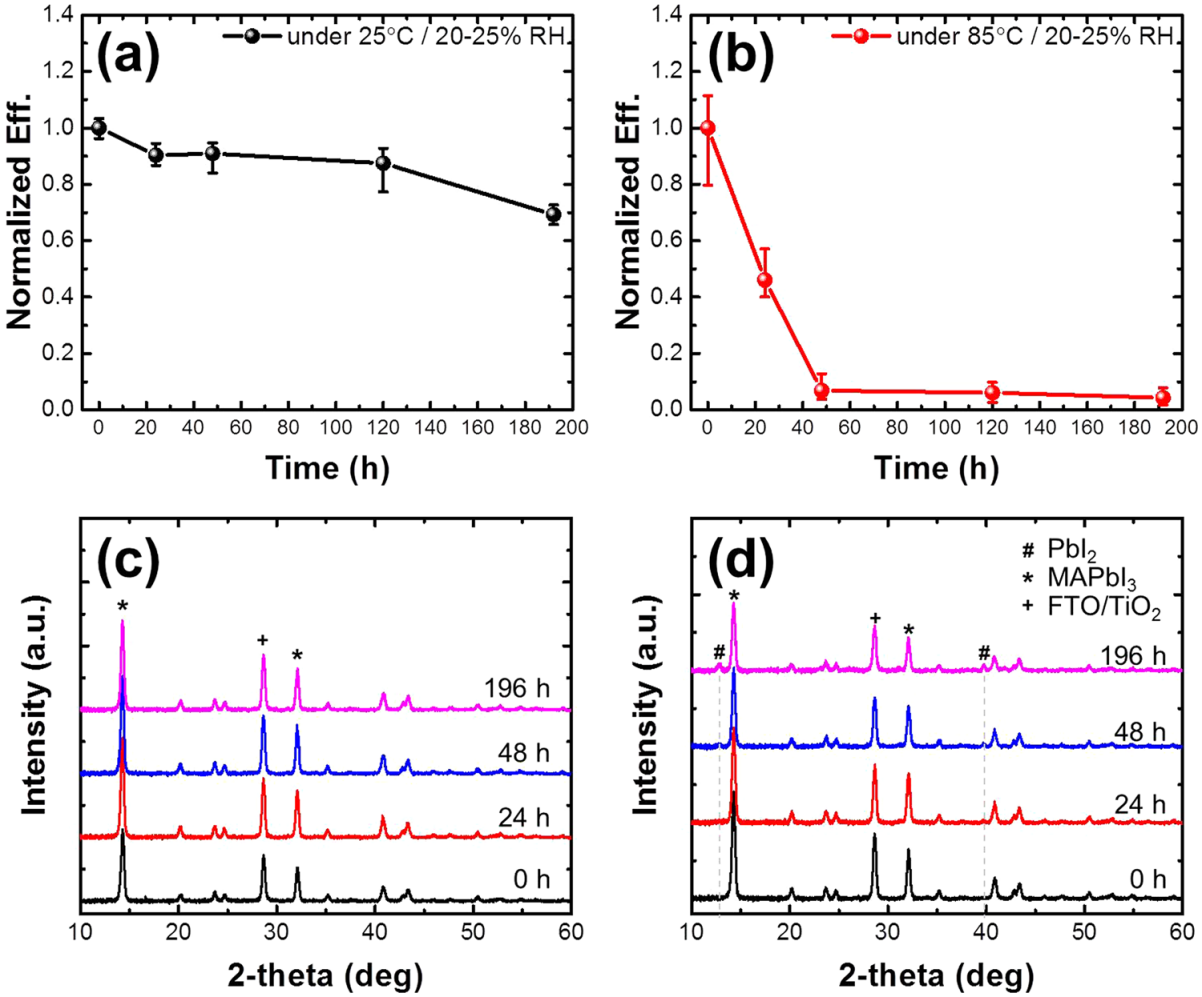
Long-term stability of MAPbI_3_ PSCs and perovskite films: power conversion efficiency at (**a**) 25 °C and (**b**) 85 °C with time and X-ray diffraction pattern of FTO/TiO_2_/MAPbI_3_/spiro-OMeTAD structure at (**c**) 25 °C and (**d**) 85 °C with time.

Several researchers have evaluated the thermal stability of MAPbI_3_ PSCs with various structures^30, 31^. However, except for the HTM-free structure, most PSCs employing an organic HTM (such as spiro-OMeTAD) degraded under thermal stress. The thermal stabilities of the PSCs developed herein were evaluated at 25 and 85 °C under ambient conditions as a function of time (Fig. 1a,b). At 25 °C, the conversion efficiency dropped by about 30% after 196 h; in contrast, at 85 °C, the conversion efficiency declined by almost 100% after 48 h. For comparison of the analysis of the phase changes that affect the solar cell efficiency, XRD patterns were acquired at 25 and 85 °C as a function of time, as shown in Fig. 1c,d. At 25 °C, the XRD pattern remained almost unchanged over time. At 85 °C, the XRD patterns remained unaffected until 48 h, at which point decomposition of the MAPbI_3_ film was observed based on the appearance of a PbI_2_ peak. In a previous study, the long-term and high-temperature stability were found to be influenced by humidity, a pinhole in the spiro-OMeTAD film, and the hygroscopic nature of Li-TFSI^11, 32^. The reports showed that exposure of the cells to high relative humidity under thermal conditions for a long period of time resulted in decomposition of the perovskite, not only because of penetration of water molecules, but also because of thermal degradation of the MAPbI_3_ perovskite film. In another study on MAPbI_3_ solar cells with remnant PbI_2_, moderate efficiencies (5–10%) were achieved relative to that of MAPbI_3_, even though PbI_2_ was fairly evenly distributed in those films based on the XRD data^33, 34^. One reported advantage of the remnant PbI_2_ was that it can act as a passivation layer for the MAPbI_3_ grains, resulting in higher solar cell efficiency rather than a drop in the efficiency^34^. However, from the XRD data used to compare the fast efficiency drop at 85 °C over time, we propose that the degradation of the solar cell performance was not only influenced by decomposition of the perovskite due to the thermal conditions, but also other factors such as degradation of the HTMs.

**Figure 2:**
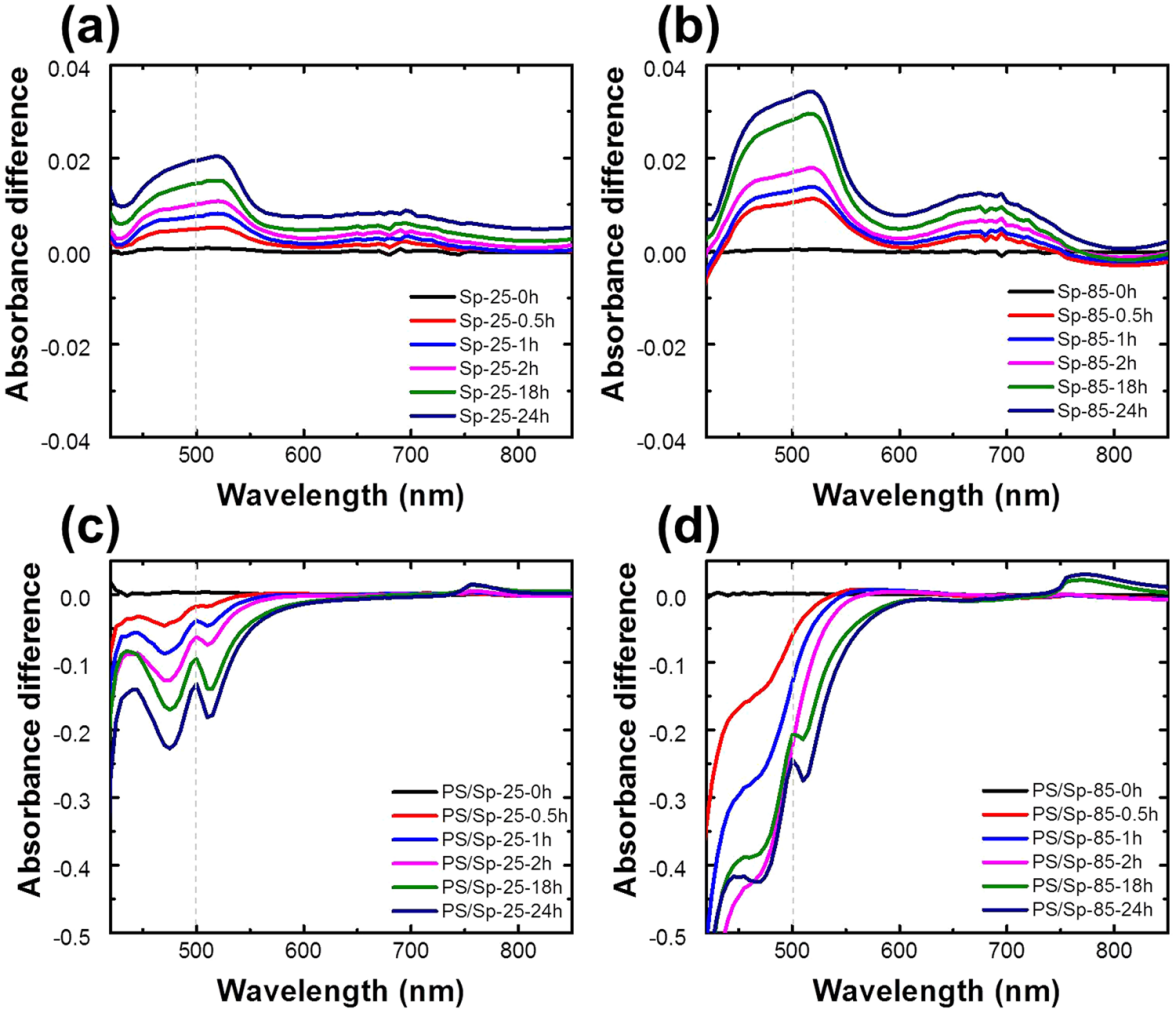
Changes in the absorbance of spiro-OMeTAD at (**a**) 25 °C and (**b**) 85 °C with time. Changes in the absorbance of MAPbI_3_/spiro-OMeTAD at (**c**) 25 °C and (**d**) 85 °C with time.

To elucidate the high-temperature (85 °C) behavior of spiro-OMeTAD, the variations of the absorbance of the spiro-OMeTAD film at 25 and 85 °C as a function of time were evaluated, as shown in Fig. 2a,b. Spiro-OMeTAD can be oxidized to spiro-OMeTAD^+^ by additives like Li-TFSI and external factors such as oxygen and light^24, 28^. Formation of spiro-OMeTAD^+^ from spiro-OMeTAD is indicated by an absorption peak in a particular wavelength region. In a previous report, when spiro-OMeTAD was oxidized to spiro-OMeTAD^+^, the optical absorption at around 500 nm increased and was accompanied by the development of electrical conductivity^24, 35^. Figure 2a shows that, at 25 °C, the absorbance around 500 nm increased as a function of time in the spiro-OMeTAD film only. Since the measurement was performed under dark and ambient conditions, the oxidation was due to oxygen and Li-TFSI. When the sample was treated at 85 °C, the oxidation reaction accelerated (Fig. 2b). The increase in the absorbance peak of spiro-OMeTAD^+^ over time indicates that spiro-OMeTAD was oxidized at both temperatures, but to a greater extent at 85 °C.

As mentioned above, spiro-OMeTAD containing MAPbI_3_ was observed to undergo enhanced oxidation under irradiation with >450-nm wavelength light^28^. This indicates that not only external conditions, but also MAPbI_3_, can affect the properties of spiro-OMeTAD. In conventional PSCs, since spiro-OMeTAD is tightly affixed to MAPbI_3_ as an HTM, spiro-OMeTAD can be influenced by MAPbI_3_ at high temperatures. The effects of temperature and time on the absorbance of MAPbI_3_/spiro-OMeTAD are summarized in Fig. 2c,d. At 25 °C, the specific absorbance peak appeared at ~500 nm after 30 min. The presence of this peak indicates that spiro-OMeTAD was oxidized at 25 °C under ambient conditions, regardless of the presence of MAPbI_3_. In contrast, at 85 °C, the absorption peak at ~500 nm did not appear until 2 h of treatment, and this specific peak was observed after 18 h when MAPbI_3_/spiro-OMeTAD was treated at 85 °C. Thus, it can be deduced that MAPbI_3_ influences the oxidation of spiro-OMeTAD at high temperatures. Since oxidation and reduction can affect the conductivity of spiro-OMeTAD, the dependence of the conductivity of spiro-OMeTAD on MAPbI_3_ at 85 °C was evaluated via a four-point probe measurement.

**Figure 3:**
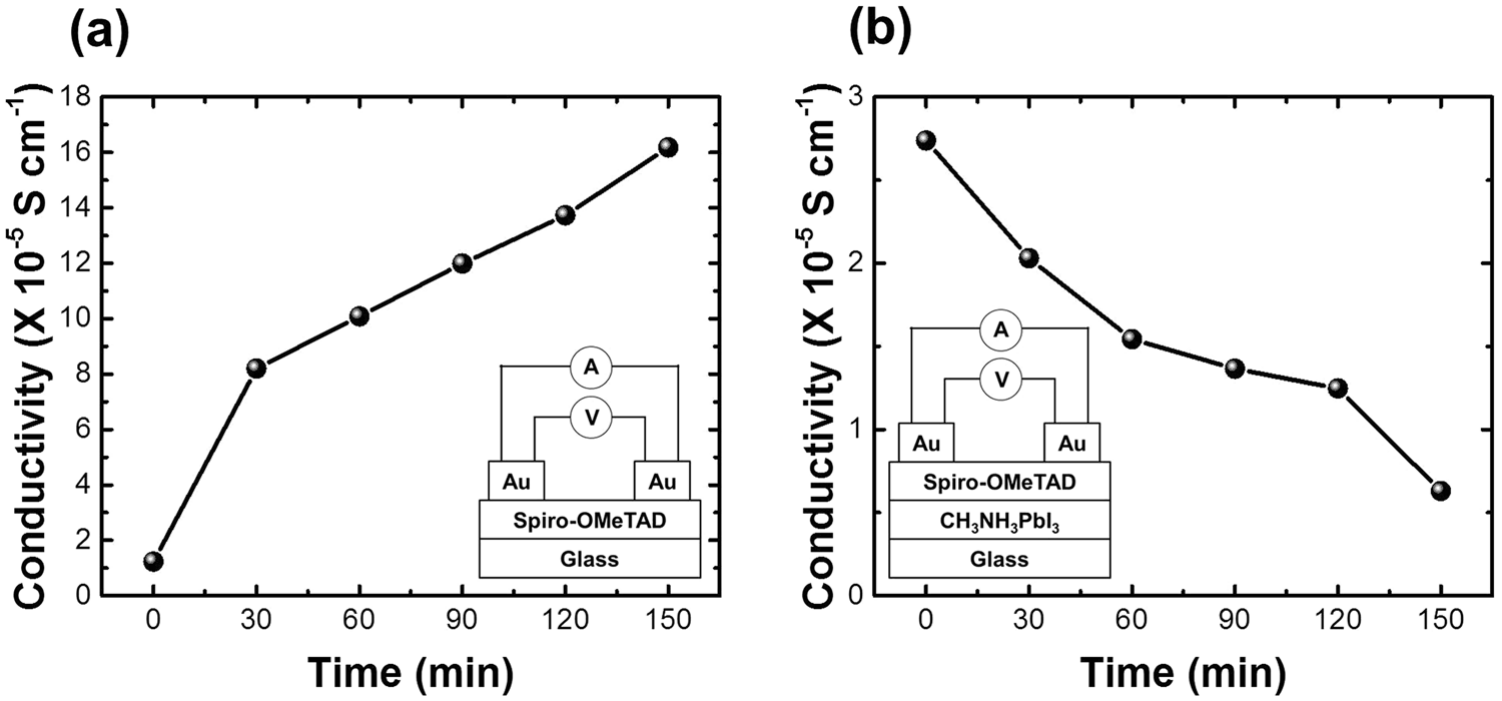
Dark conductivity of (**a**) spiro-OMeTAD and (**b**) MAPbI_3_/spiro-OMeTAD at 85 °C with time. Four-point probe measurement was used for the conductivity analysis. Inset shows structures for the measurement of conductivity of spiro-OMeTAD, glass/spiro-OMeTAD/Au, and glass/MAPbI_3_/spiro-OMeTAD/Au.

As shown in the inset in Fig. 3a,b, glass/spiro-OMeTAD/Au and glass/MAPbI_3_/spiro-OMeTAD/Au structures were used for the measurements. The initial difference in values was due to the different surface morphologies of glass and MAPbI_3_ despite the same spin coating condition. Since the thickness of spiro-OMeTAD was about 200–250 nm in this study, the conductivity of 1–3 × 10^−5^ Scm^−1^ for spiro-OMeTAD was similar to previous results^28, 36^. Figure 3a,b show the variation of the conductivity of spiro-OMeTAD with time at 85 °C for each structure. In the spiro-OMeTAD film, the conductivity of spiro-OMeTAD increased upon treatment at 85 °C, whereas the conductivity of spiro-OMeTAD in the MAPbI_3_/spiro-OMeTAD structure decreased. Comparison of this absorption data with that of spiro-OMeTAD (Fig. 2b) revealed that the conductivity of spiro-OMeTAD without MAPbI_3_ increased because of oxidation of spiro-OMeTAD at 85 °C. On the other hand, the decreased conductivity of MAPbI_3_/spiro-OMeTAD indicates that oxidation of spiro-OMeTAD was hindered because of an interfacial reaction between MAPbI_3_ and spiro-OMeTAD at 85 °C (Fig. 3b). As mentioned above, a decrease of the conductivity of spiro-OMeTAD due to incorporation of MAI was reported^29^. A previous study reported that, since iodine ions, such as those derived from MAI, are highly coordinating, the decreased polaronic band resulted in reduction of spiro-OMeTAD^+^. Iodide ions can move easily because of their very low ion migration energy (0.16–0.43 eV)^15, 37, 38^. At high temperatures, the mobile iodide ions easily diffuse to MAPbI_3_ as well as nearby layers, such as the spiro-OMeTAD layer, thereby changing the electrical properties of spiro-OMeTAD. Similar conductivity behaviors of spiro-OMeTAD were also observed with increasing temperature in the same structure (Figure S2). With increasing temperature, the conductivity of spiro-OMeTAD without MAPbI_3_ increased, whereas that in the MAPbI_3_/spiro-OMeTAD structure did not. Since the extent of ion migration is proportional to temperature, the decreased conductivity reflects the possibility of the migration of ions such as I^−^ and MAI with increasing temperature. Based on these results, we proposed that thermally diffused I^−^ and MAI negatively influence spiro-OMeTAD at high temperatures.

When spiro-OMeTAD is not sufficiently oxidized, PSCs exhibit very low conversion efficiencies^39^. Oxidation of spiro-OMeTAD not only increased the conductivity, but also increased the charge collection because of a shift of the energy level. Thus, to promote oxidation of spiro-OMeTAD, Li-TFSI was added to spiro-OMeTAD. The EQEs of the thermally degraded PSCs were compared with that of the pristine PSCs without Li-TFSI (Figure S3). In the case of the PSCs without Li-TFSI, because spiro-OMeTAD was not sufficiently oxidized, a low EQE response between 500–800 nm was obtained. The EQE curve of the degraded PSCs was similar to that of the PSCs without Li-TFSI (i.e., not sufficiently oxidized). Thus, this result indicates that, although MAPbI_3_ was not excessively decomposed, the efficiency of the PSCs may be reduced because of reduction of spiro-OMeTAD, leading to decreased hole collection.

**Figure 4:**
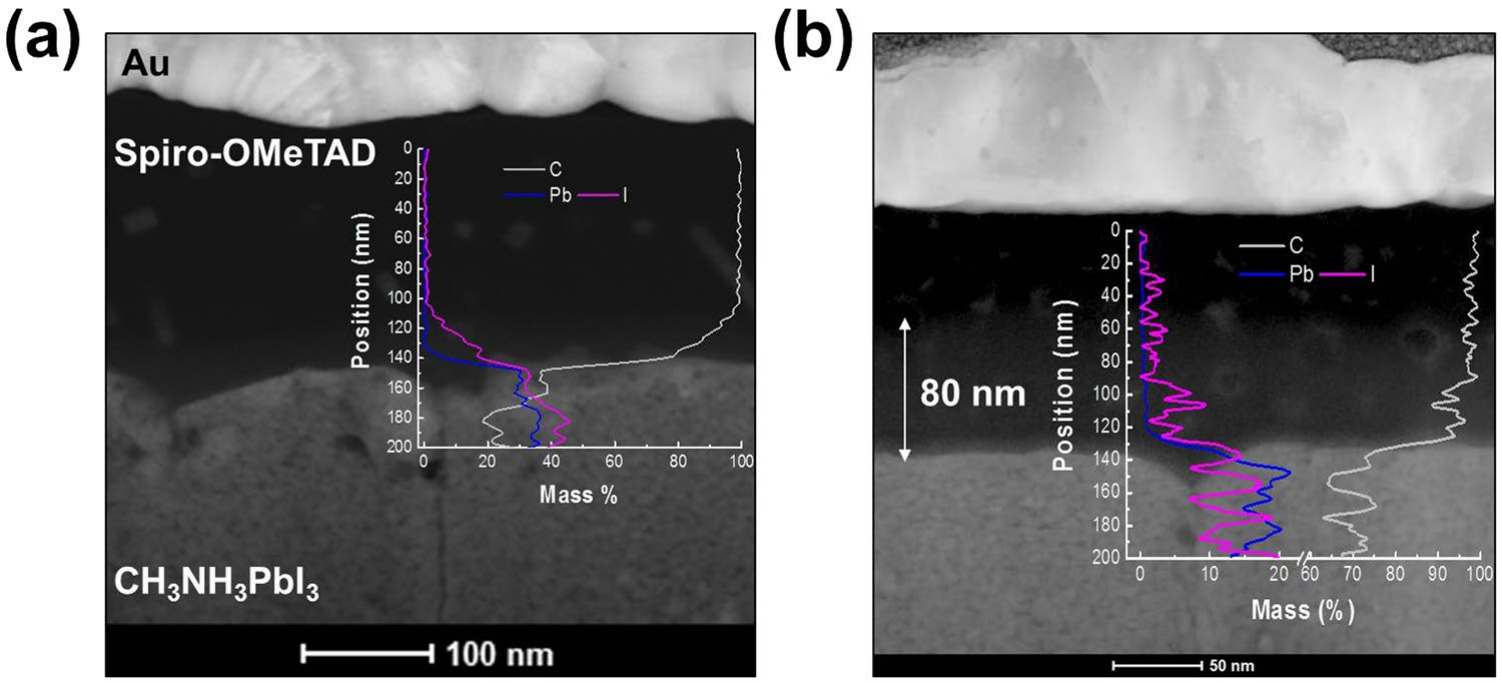
High-angle annular dark-field (HAADF) scanning transmission electron microscopy (TEM) images showing cross-sectional views of the MAPbI_3_/spiro-OMeTAD interface: (**a**) pristine and (**b**) thermally stressed at 85 °C for 2 h. Inset shows EDX line maps for lead and iodine. Contrast images and EDX display the presence of iodine.

Recently, transmission electron microscopy (TEM) and energy-dispersive X-ray spectroscopy (EDX) were used to monitor the effects of increasing the temperature up to 300 °C (using *in-situ* TEM) on spiro-OMeTAD^40^. Iodide ions began diffusing into spiro-OMeTAD at 175 °C because of the high mobility of the iodide ions. These mobile iodide ions originate from MAPbI_3_ or excess MAI present during formation of the perovskite film. In this study, the iodide ions were also observed via TEM analysis of the MAPbI_3_/spiro-OMeTAD interface (Fig. 4). EDX line mapping of the interface is displayed in the inset. In contrast with the TEM and EDX data, it is evident that iodine was present in spiro-OMeTAD: iodine was present in the fresh sample of spiro-OMeTAD to a depth of <20 nm at the MAPbI_3_/spiro-OMeTAD interface (Fig. 4a). This iodine may be derived from several sources such as dissolution of the MAPbI_3_ surface during spin-coating of spiro-OMeTAD or disconnection between MAI and PbI_2_ during annealing of the MAPbI_3_ film. Interestingly, migration of iodine to a depth of ~80 nm into spiro-OMeTAD was observed only with treatment at 85 °C for 2 h (Fig. 4b). This iodine migration at 85 °C was elucidated and confirmed by time-of-flight secondary ion mass spectrometry (TOF-SIMS, Figure S4).

The origin of the migratory iodide ions remained unconfirmed. Iodine moved along the grain boundary (GB) of the MAPbI_3_ grains under an electric field^41^. The iodine and lead profiles of a MAPbI_3_ GB sample degraded at 85 °C are shown in Figure S5. While the atomic ratio of lead was constant, that of iodine in the GB declined. Furthermore, the atomic ratio of iodine decreased to a greater extent while moving toward spiro-OMeTAD. Since iodide ions migrate faster in the GB than in bulk MAPbI_3_^41^, the iodide ions in the MAPbI_3_ films can also migrate at high temperatures. Therefore, we propose that the migratory iodide ions were derived from mobile ions in the MAPbI_3_ films.

**Figure 5:**
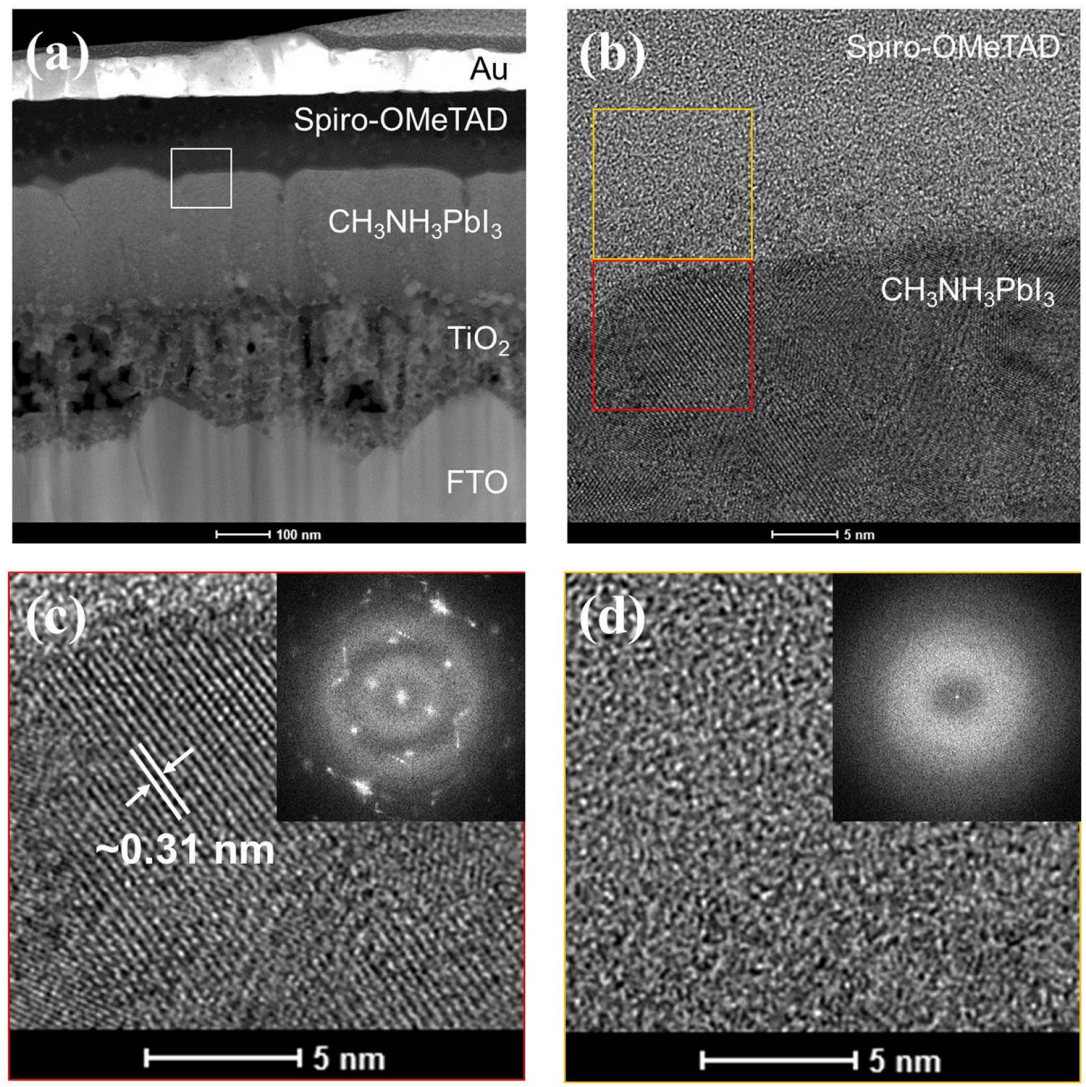
(**a**) TEM micrograph of FTO/TiO_2_/MAPbI_3_/spiro-OMeTAD/Au sample. (**b**) High resolution TEM image of MAPbI_3_/spiro-OMeTAD interface shown in (**a**): white square; (**c**,**d**) highly magnified TEM image of MAPbI_3_ and spiro-OMeTAD shown in (**b**): red square and yellow square, respectively. Inset images show the fast Fourier transform pattern.

For more detailed analyses, the thermally degraded PSCs were characterized by high-resolution TEM (HRTEM) and fast Fourier-transform (FFT) analysis (Fig. 5). Figure 5b–d show TEM images of the MAPbI_3_/spiro-OMeTAD interface, MAPbI_3_ proximal to spiro-OMeTAD, and spiro-OMeTAD proximal to MAPbI_3_, respectively. The lattice parameter of MAPbI_3_ was determined to be about 0.31 nm, which is similar to the (004) or (220) tetragonal phase of MAPbI_3_^42^. Although iodine partially diffused into spiro-OMeTAD, the crystal lattice of MAPbI_3_ did not change with thermal treatment at 85 °C for 2 h. There were no observations of changes to the crystal lattice and diffraction pattern due to iodine diffusion in spiro-OMeTAD (Fig. 5d). Spiro-OMeTAD crystallizes at 100 °C, resulting reduced conductivity^43^. However, no spiro-OMeTAD crystallization was observed by FFT and XRD in this study (Figure S6). Since the diffraction pattern of spiro-OMeTAD was not observed, it is evident that the iodine was present in spiro-OMeTAD as ions rather than precipitates. Thus, iodine as an ion reacts easily with spiro-OMeTAD, resulting in reduction of spiro-OMeTAD^+^. Since ion migration is fast in organic materials, such as small molecules, this migration may be the origin of the efficiency drop of PSCs at high temperatures within a short time period rather than decomposition of the perovskite. As supporting evidence, it was reported that thermally stable PSCs with HTM-free structures (i.e., without an organic HTM) experienced a <10% efficiency drop^30^. These researchers developed PSCs with an m-TiO_2_/ZrO_2_/MAPbI_3_ structure without an organic HTM, thereby achieving greatly enhanced thermal stability. Furthermore, several structures with a barrier film between MAPbI_3_ and spiro-OMeTAD have been reported^12, 44^. Although the role of the barrier film was to protect the layers from moisture, it was proposed that the film could also block the diffusion of iodide ions.

**Figure 6:**
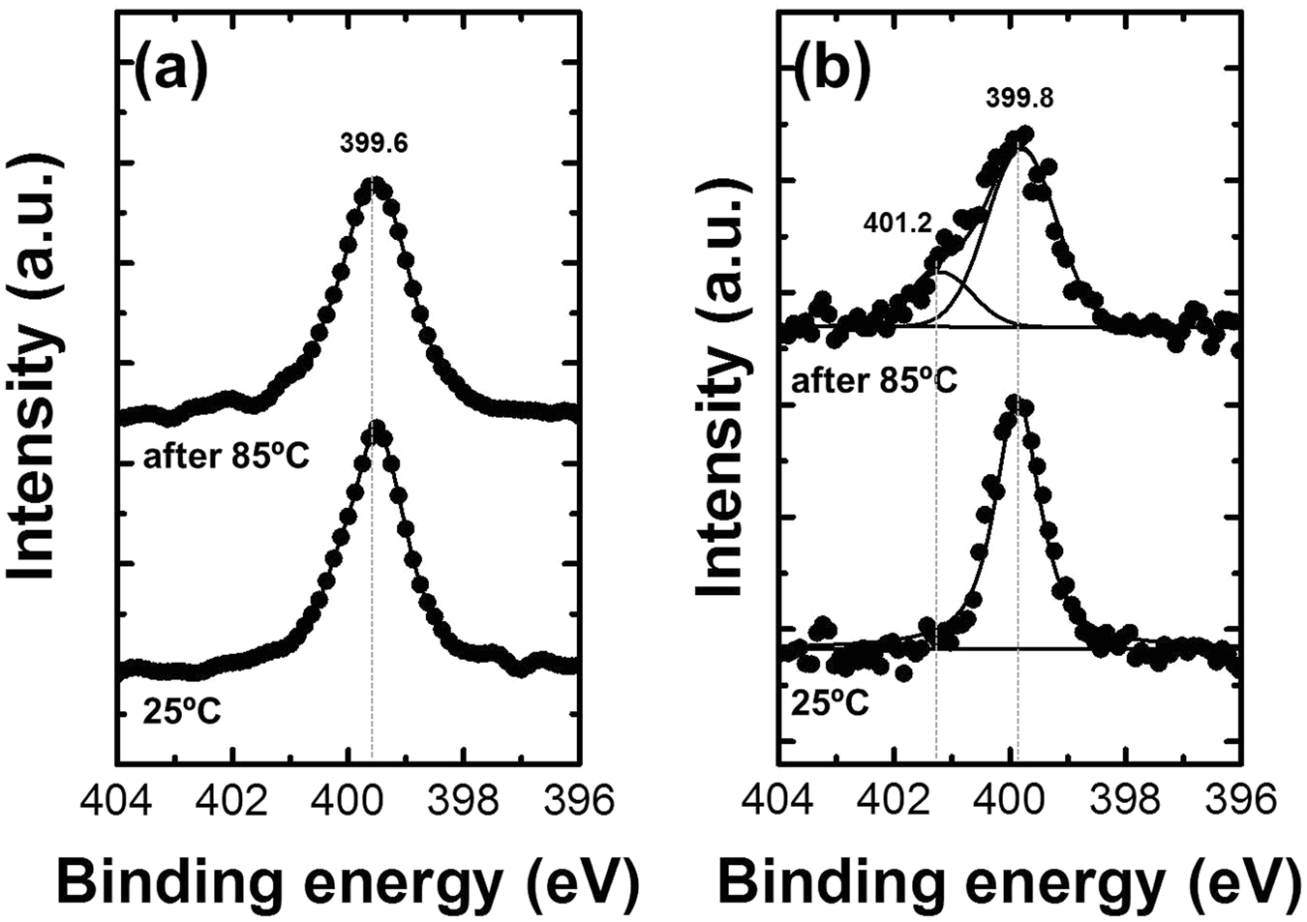
N 1s XPS narrow scan spectra of (**a**) spiro-OMeTAD only and (**b**) MAPbI_3_/spiro-OMeTAD as-prepared (25 °C) and after thermal treatment (85 °C). XPS data were acquired from the spiro-OMeTAD side.

To verify the changes in the chemical bonding of spiro-OMeTAD, the binding energies were assessed by X-ray photoelectron spectroscopy (XPS) at varying temperatures (Fig. 6). The results for spiro-OMeTAD and MAPbI_3_/spiro-OMeTAD samples were compared after treatment at 25 and 85 °C. A N 1s species with an energy of 399.8 eV was observed and corresponds to C–N in spiro-OMeTAD and the imide group in Li-TFSI^32, 45^.

After treatment at 85 °C, N 1s of spiro-OMeTAD remained and a peak at 401.2 eV appeared in the MAPbI_3_/spiro-OMeTAD structure. This binding energy corresponds to amine groups such as CH_3_NH_2_^46^. It is thought that the diffused MAI separated into CH_3_NH_2_ and HI at 85 °C^10, 47^. Thus, not only I^−^ but also MA^+^ from MAPbI_3_ diffused under thermal conditions. In contrast with the N 1s peaks, the other C, Pb, and I bonds changed only marginally (Figure S7).

From the TEM, EDX, and XPS data, MAI-poor region may form at the MAPbI_3_/spiro-OMeTAD interface. Since the MAI-poor film in MAPbI_3_ acts as an n-type film because of an increase in the Fermi level^48^, energy barrier bands formed at the MAPbI_3_/spiro-OMeTAD interface because of energy band bending. When a hole is collected during operation, the energy barriers block and rectify the motion of the hole. Thus, these energy barriers can also hinder the collection of charge carriers, thereby decreasing the solar cell performance.

**Figure 7:**
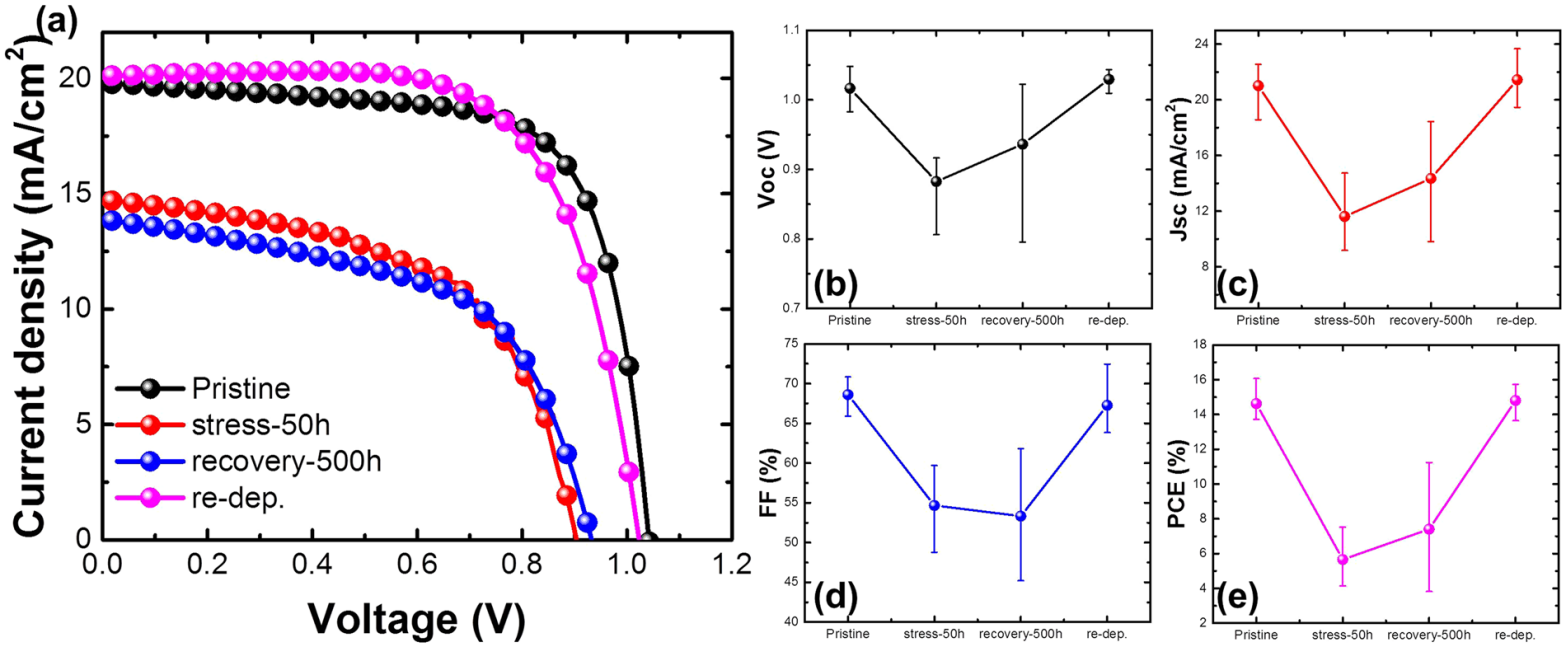
Step-by-step procedures for the re-deposition of spiro-OMeTAD: 1) pristine, 2) after thermal treatment at 85 °C for 50 h, 3) after recovery at 25 °C for 500 h, and 4) after removal of Au and spiro-OMeTAD followed by re-deposition of spiro-OMeTAD. (**a**) Representative *I–V* curves according to the spiro-OMeTAD conditions. Average values of seven cells with procedures plotted to (**b**) *V*_*oc*_, (**c**) *J*_*sc*_, (**d**) *FF*, and (**e**) PCE.

In order to confirm the effect of spiro-OMeTAD degradation, the PSC performances were compared depending on the spiro-OMeTAD conditions (Fig. 7). The following procedure was used for re-deposition of spiro-OMeTAD: (1) I–V measurements of the pristine condition; (2) I–V measurements after thermal treatment at 85 °C for 50 h under inert Ar; (3) I–V measurements after recovery at 25 °C for 500 h under inert Ar; and (4) I–V measurements after removal of Au and spiro-OMeTAD and after re-deposition of spiro-OMeTAD and Au. Initially, the average efficiency of the PSCs was 15%, and, despite a 500 h recovery time, the PSC efficiency remained around 7% after thermal treatment. However, after re-deposition of spiro-OMeTAD and Au, the PSC performance almost completely recovered to the initial values. These results indicate that spiro-OMeTAD was degraded at 85 °C rather than MAPbI_3_. In other words, the main reason for the degradation of the PSC performance at 85 °C was the degradation of spiro-OMeTAD. This degradation and recovery behavior was observed regardless of the atmosphere, i.e., ambient air or inert Ar (Figure S8). Because thermal diffusion of ions is not dependent on the atmosphere, the rapid efficiency drop at 85 °C was attributed to spiro-OMeTAD degradation caused by ion diffusion rather than decomposition of MAPbI_3_.

To prevent ion diffusion, the stability of m-TiO_2_/MAPbI_3_/MoO_x_/spiro-OMeTAD with a <10 nm MoO_x_ interface layer was evaluated at 85 °C (Figure S9). The results showed slight moderation of the thermal degradation. The MoO_x_-inserted cell showed a lower initial efficiency because MoOx is not suitable as an interface layer between MAPbI_3_ and spiro-OMeTAD with respect to electrical properties (Table S1). Although MoO_x_ is not an ideal diffusion barrier, the reduction in degradation was attributed to hindered ion migration. A reduction in iodine diffusion was confirmed by TOF-SIMS (Figure S10).

Ion migration into spiro-OMeTAD degrades the electrical properties of spiro-OMeTAD through reduction and decreases charge-carrier collection through the formation of energy barriers. Because this kind of ion migration can occur during operation outdoor or in other external environments, the compositional and structural design of PSCs must be optimized to achieve long-term stability.

In this study, to determine the cause of the reduction in efficiency of spiro-OMeTAD–based PSCs upon thermal exposure at 85 °C for a short time period, we investigated the thermal behavior of spiro-OMeTAD and MAPbI_3_/spiro-OMeTAD films. The results showed that spiro-OMeTAD was highly oxidized at 85 °C; in contrast, the oxidation of spiro-OMeTAD in MAPbI_3_/spiro-OMeTAD was hindered by diffusion of iodide ions from MAPbI_3_ at temperatures above room temperature, resulting in decreased conductivity of spiro-OMeTAD. Iodide ions diffuse easily into spiro-OMeTAD and act as reducing agents for spiro-OMeTAD at elevated temperatures. Since CH_3_NH_2_ was detected on the surface of spiro-OMeTAD, it was concluded that iodide ions migrated in the form of MAI. The observed re-deposition of spiro-OMeTAD rather than degraded spiro-OMeTAD suggests that the main reason for the degradation of PSCs at 85 °C was degradation of spiro-OMeTAD rather than decomposition of MAPbI_3_. This thermally activated ion diffusion may occur during outdoor operation of PSCs and can decrease the conversion efficiency. Therefore, the compositional and constructional design of perovskites or PSCs must be considered to enhance the thermal and overall stability of PSCs.

## Electronic supplementary material

Surpprting information
