# Supplementary material for: Relationship between ion migration and interfacial degradation of CH3NH3PbI3 perovskite solar cells under thermal conditions
Source: Sci Rep. 2017 Apr 26;7:1200. doi: 10.1038/s41598-017-00866-6 (PMC5430925; doi:10.1038/s41598-017-00866-6)
Supplement: Supplementary file 1 — Surpprting information [file 41598_2017_866_MOESM1_ESM.doc]

**Supporting Information**

Relationship between ion migration and interfacial degradation of CH_3_NH_3_PbI_3_ perovskite solar cells under thermal conditions

Seongtak Kim, ^1^ Soohyun Bae, ^1^ Sang-won Lee, ^1^ Kyungjin Cho, ^1^ Kyung Dong Lee, ^1^ Hyunho Kim, ^1^ Sungeun Park, ^1^ Guhan Kwon, ^2^ Sehwon Ahn, ^2^ Heon-Min Lee, ^2^ Yoonmook Kang, ^*,3^ Hae-Seok Lee,^*, 3^ and Donghwan Kim^*,1^

^1^ Department of Materials Science and Engineering, Korea University, 145 Anam-ro, Seongbuk-gu, Seoul 02841, Republic of Korea

^2^ Materials & Devices Advanced Research Institute, LG Electronics, 38 Baumoe-ro, Seocho-gu, Seoul 06763, Republic of Korea

^3^ KU·KIST Green School Graduate School of Energy and Environment, Korea University, 145 Anam-ro, Seongbuk-gu, Seoul 02841, Republic of Korea

KEYWORDS: perovskite solar cells, methylammonium lead iodide, spiro-OMeTAD, ion migration, thermal stability, efficiency recovery, re-deposition

**METHODS**

**Materials and Device Fabrication**

Lead(II) iodide (99.9958%) was purchased from Alfa Aesar, 18NR-T and MAI were purchased from Dyesol, and 2,2',7,7'-tetrakis(*N,N*-di-*p*-methoxyphenylamino)-9,9'-spirobifluorene (spiro-OMeTAD) was obtained from Lumtec. All other materials were purchased from Sigma-Aldrich. Patterned FTO glass substrates (7 Ω·sq^−1^) were consecutively cleaned with acetone, ethanol, and isopropanol for 10 min. The TiO_2_ compact layer (c-TiO_2_) was deposited onto the FTO substrates by spin-coating of a TiO_2_ precursor solution (0.15 M titanium diisopropoxide bis (acetyleacetonate) in 1-butanol) followed by drying at 125 °C for 5 min on a hot plate. A mesoporous TiO_2_ (m-TiO_2_) layer was deposited onto the c-TiO_2_ substrates by spin-coating of a mixed solution (18NR-T paste, terpineol, and ethyl alcohol) followed by drying at 100 °C for 1 min on a hot plate. After spin-coating, the substrates were annealed at 550 °C for 1 h in air. The MAPbI_3_ solution for the one-step deposition comprised 50 wt% 1:1:1 MAI, PbI_2_, and DMSO in DMF. MAPbI_3_ perovskite layers were deposited onto the m-TiO_2_ substrates by spin-coating: diethyl ether was dropped onto the substrate during the spin-coating process. The HTM solution was prepared by mixing 72.3 mg of spiro-OMeTAD in 1 mL of chlorobenzene with 28.8 µL of 4-tert-butylpiridine and 17.5 µL of Li-bis(trifluoromethanesulfonyl) imide (Li-TFSI) solution (520 mg in 1 mL of acetonitrile); this solution was used to apply a coating using a spin coater. Finally, the contacts were deposited by thermal evaporation of 100 nm of Au in a vacuum chamber. To generate the m-TiO_2_/MAPbI_3_/MoO_x_/spiro-OMeTAD/Au structured sample, a 10 nm MoO_x_ layer was deposited by thermal evaporation at 0.2 Å∙s^−1^. The cells were not encapsulated.

**Device Characterization**

The phases of the perovskite films were observed by XRD (Rigaku, SmartLab). The top view of the perovskite films was observed using a field emission scanning electron microscope (FEI Quanta 250 FEG). The absorption spectra of the perovskite films were acquired using an ultraviolet-visible–near infrared (UV-Vis-NIR) spectrophotometer (V-670 UV-VIS-NIR spectrophotometer, JASCO). To evaluate the effect of MAPbI_3_ on the conductivity of spiro-OMeTAD, glass/spiro-OMeTAD/Au and glass/MAPbI_3_/spiro-OMeTAD/Au structures with an inter-electrode distance of 1 mm were used for the four-point probe test. The photocurrent *I*–*V* performance (light *I*–*V*) of the PSCs was measured using a Keithley 2400 source meter with an AM1.5G one-sun solar simulator (WACOM WXS-155S-10 class AAA). The light *I*–*V* curves were obtained using a delay time of 200 ms at each point in the reverse scan from the open circuit voltage to short circuit current (*V_oc_* to *J_sc_*). The EQE curves were measured using a monochromatic light probe at 300–800 nm (QEX10, PV measurements). The chemical bonds of specific atoms were evaluated by XPS (ULVAC-PHI, X-TOOL) to observe changes in the chemical bonding of the spiro-OMeTAD surface. The cross-sectional images and components were analyzed using a transmission electron microscope (Talos F200 X, FEI) and EDX, respectively. Time-of-flight secondary ion mass spectrometry 5 (TOF-SIMS 5, ION-TOF) was used with Cs^+^ primary ions (current: 10 KeV, 25 nA, raster size: 200 µm × 200 µm) for sputter conditions and Bi_3_^+^ primary ions (current: 25 KeV, 0.5 pA, raster size: 50 µm × 50 µm) for analysis.

For applying thermal stress, the temperature was controlled using a hot plate and checked using a thermocouple (k-type) and thermometer (Testo-925). Ambient conditions were maintained with 20–25% relative humidity in a homemade dark box. Inert Ar conditions were maintained in an Ar-filled glove box (<0.3 ppm moisture).

**Figures**


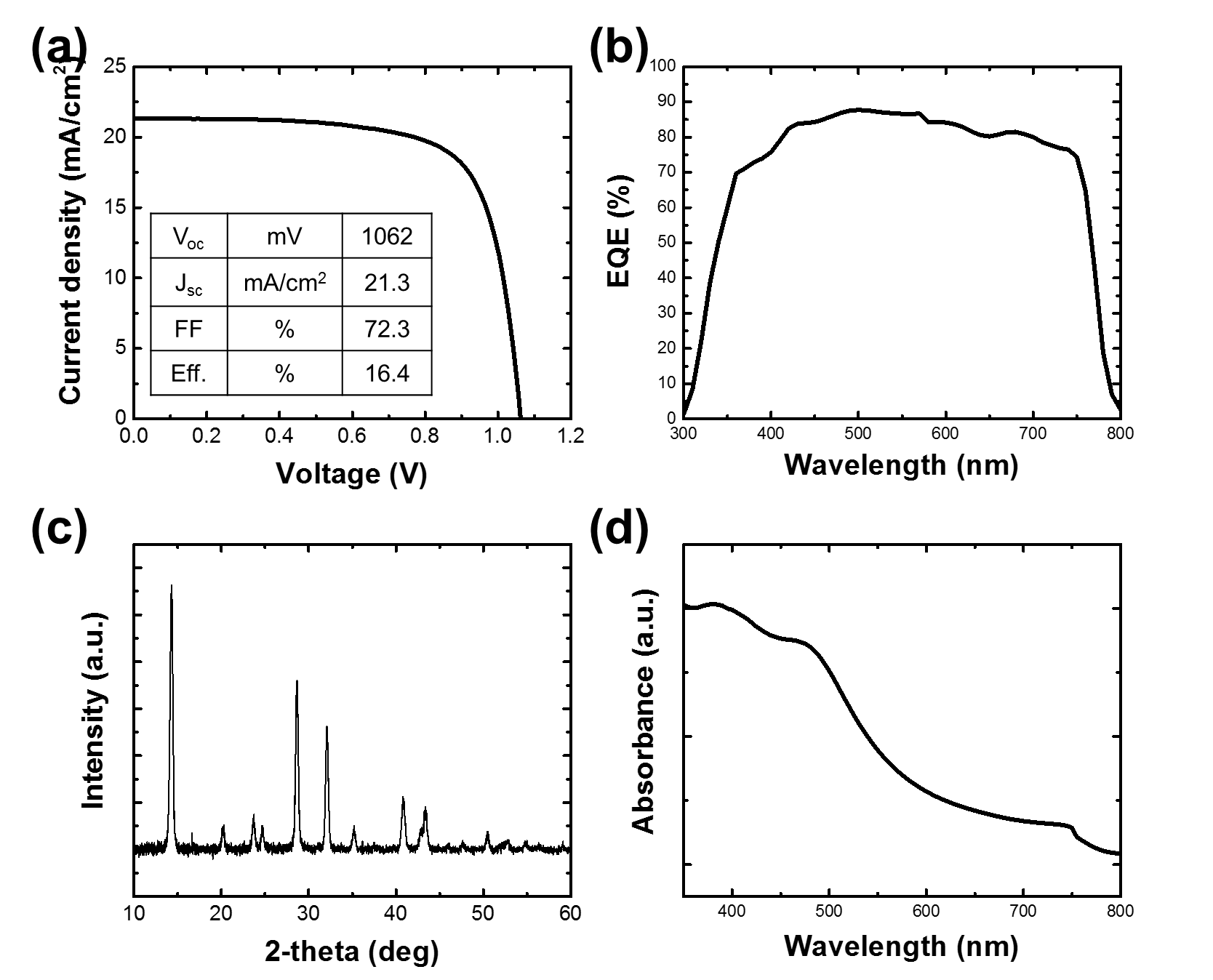


**Figure S1.** Characterization of the perovskite film and PSCs: (a) Light *I–V* curve and parameters of the prepared MAPbI_3_ perovskite solar cell. (b) EQE curve of the MAPbI_3_ solar cell. (c) X-ray diffraction pattern and (d) absorbance of the FTO/TiO_2_/CH_3_NH_3_PbI_3_ (MAPbI_3_) structure.


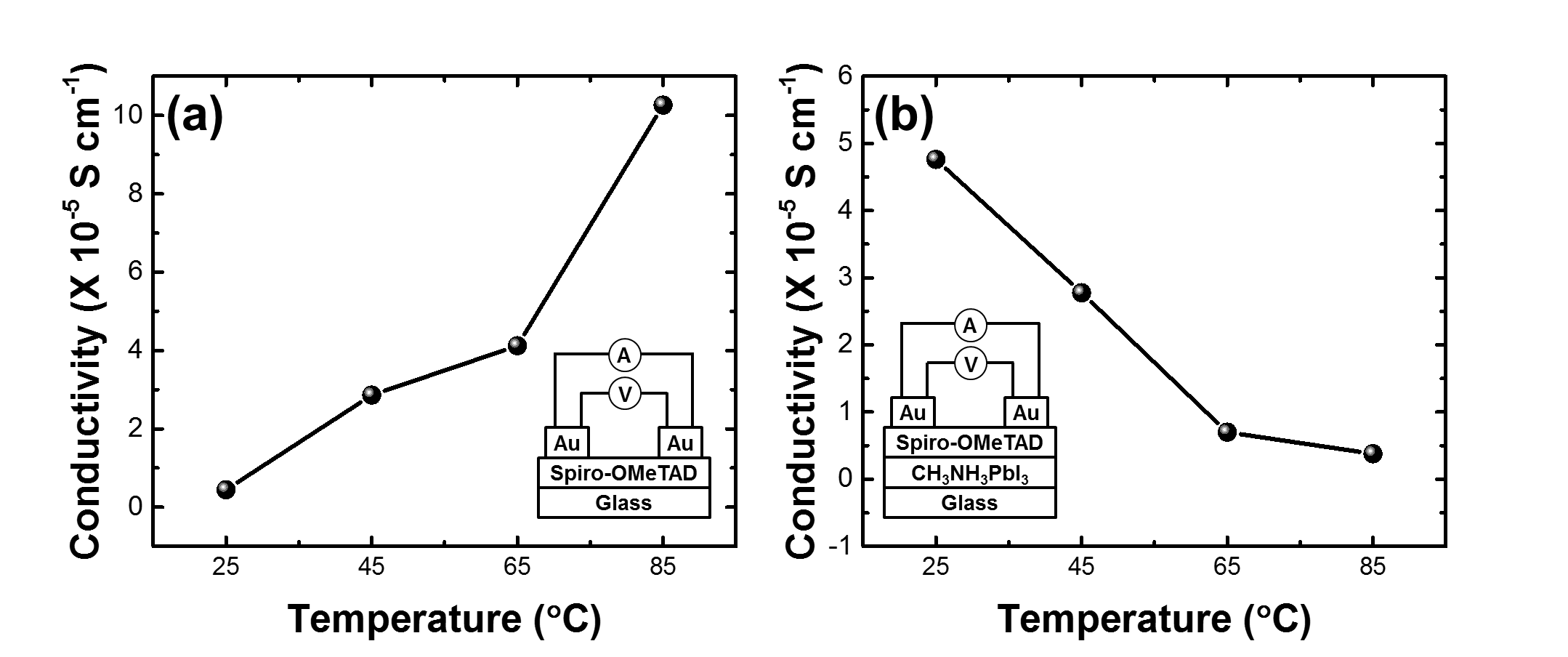


**Figure S2.** (a) Dark conductivity of spiro-OMeTAD and (b) MAPbI_3_/spiro-OMeTAD with increasing temperature. Before the measurements, the samples were maintained at each temperature for 30 min. Four-point probe measurements were used for the conductivity measurements. Glass/spiro-OMeTAD/Au and glass/MAPbI_3_/spiro-OMeTAD/Au structures were used.


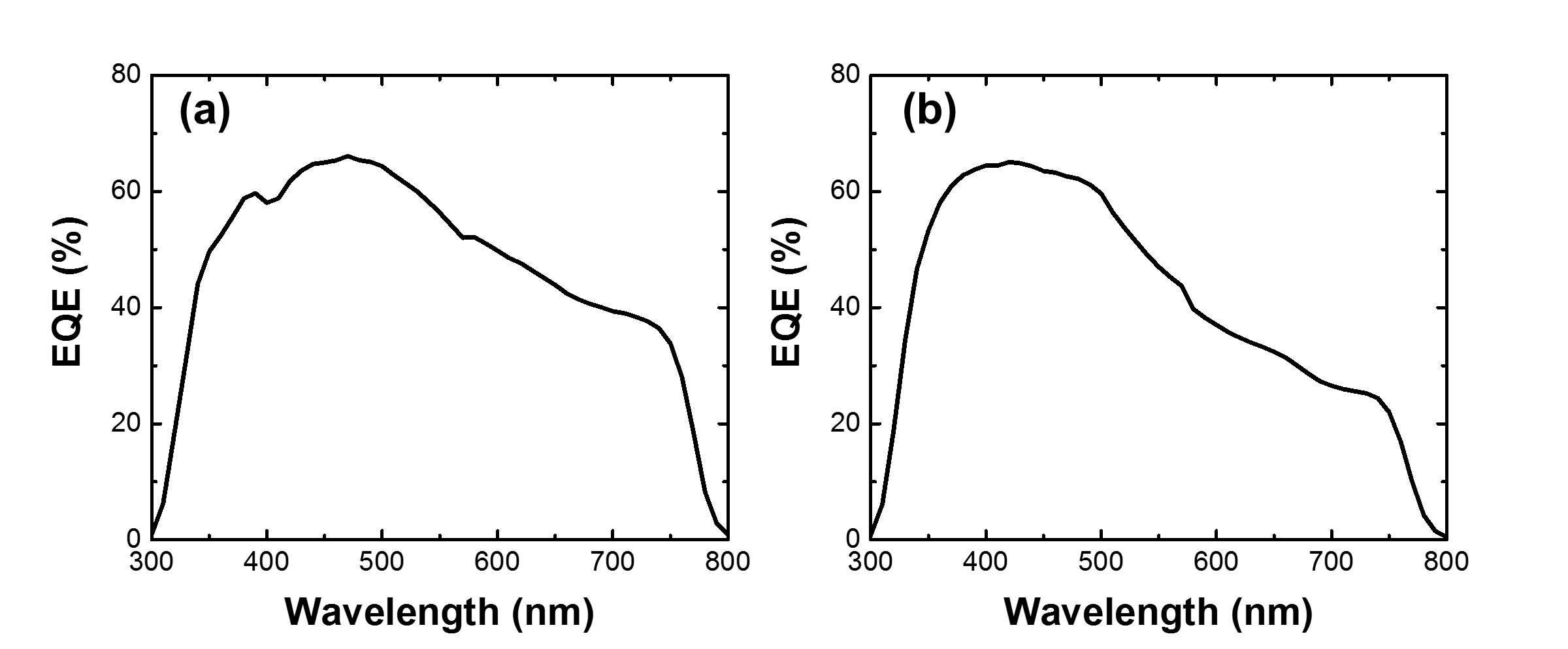


**Figure S3.** EQE curves of (a) TiO_2_/MAPbI_3_/spiro-OMeTAD/Au–structured PSCs after thermal treatment at 85 °C for one day and (b) the as-prepared structure without Li-TFSI additive.


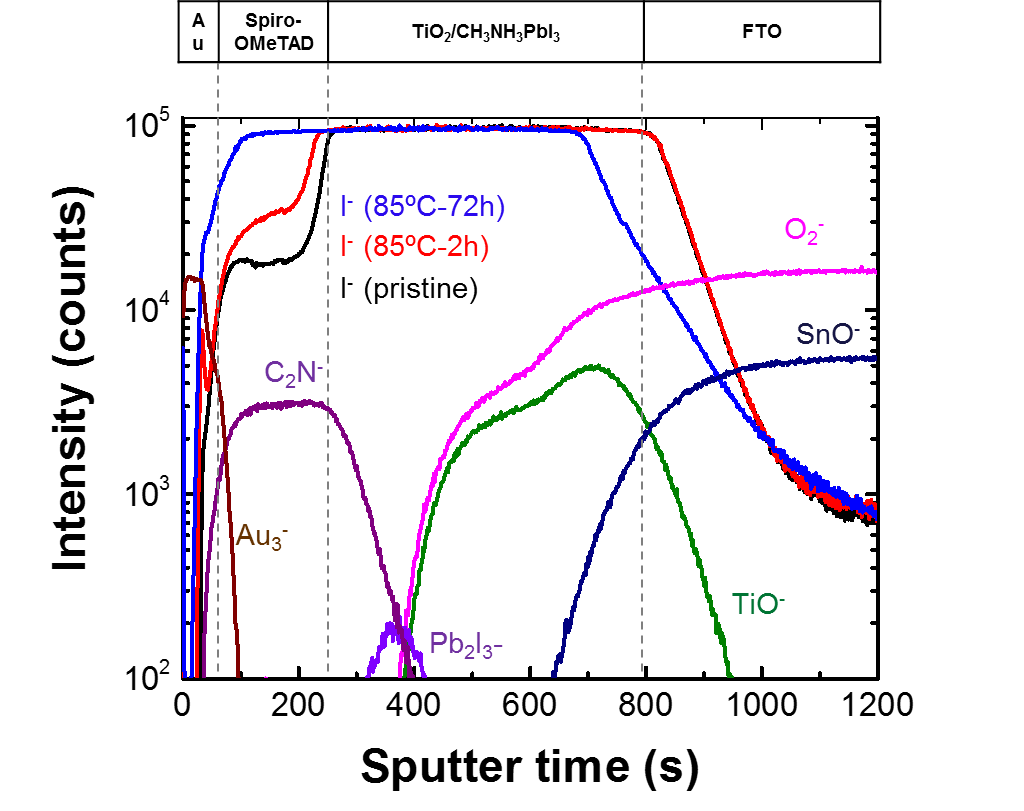


**Figure S4.** TOF-SIMS depth profiles of the concentrations of selected species. The profiles are for pristine PSCs and those thermally treated (85 °C) for 2 and 72 h.


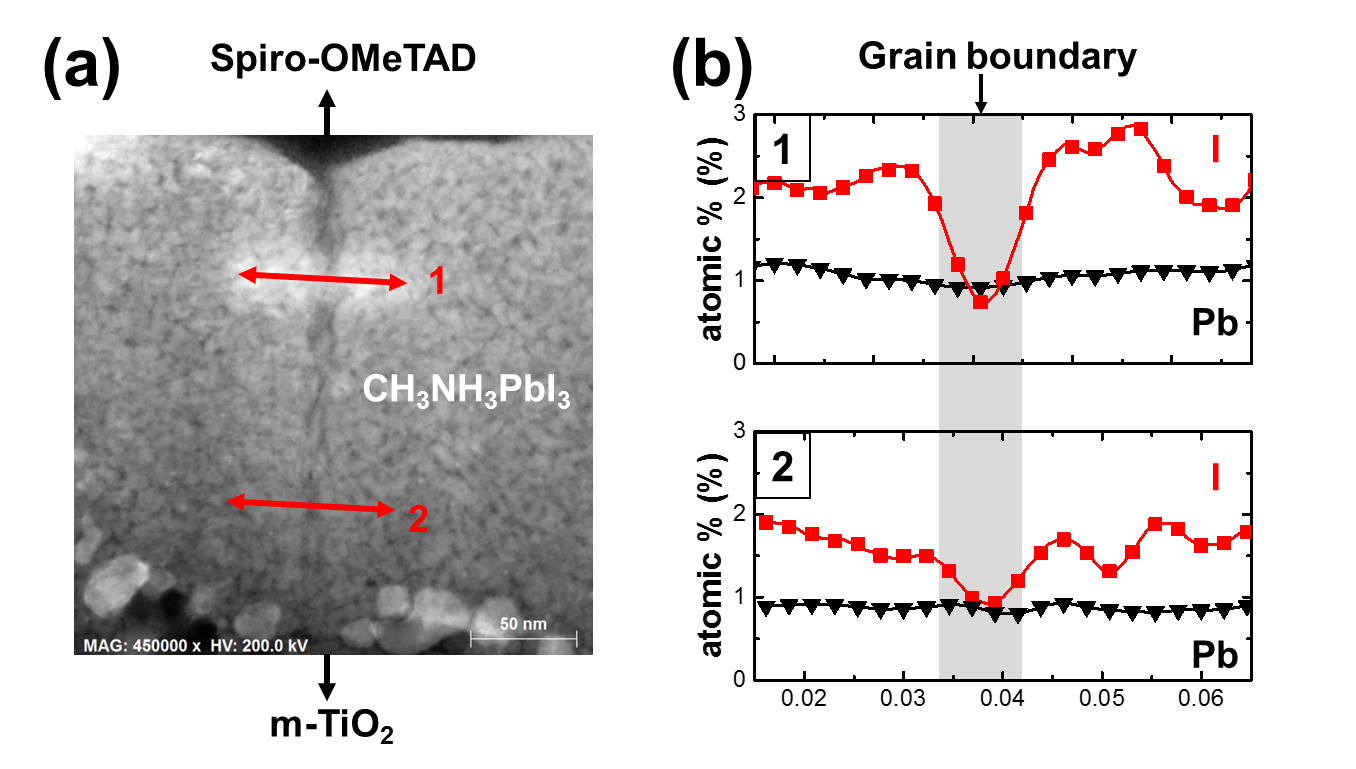


**Figure S5.** TEM and EDX analysis of the redistribution of Pb and I at the GB after thermal treatment. (a) TEM image of the MAPbI_3_ polycrystalline structure with GBs with m-TiO_2_ and spiro-OMeTAD. (b) Atomic ratio of I to Pb obtained from EDX line scanning, where the number indicates the scanning area in (a). Grey rectangles indicate the GB area.


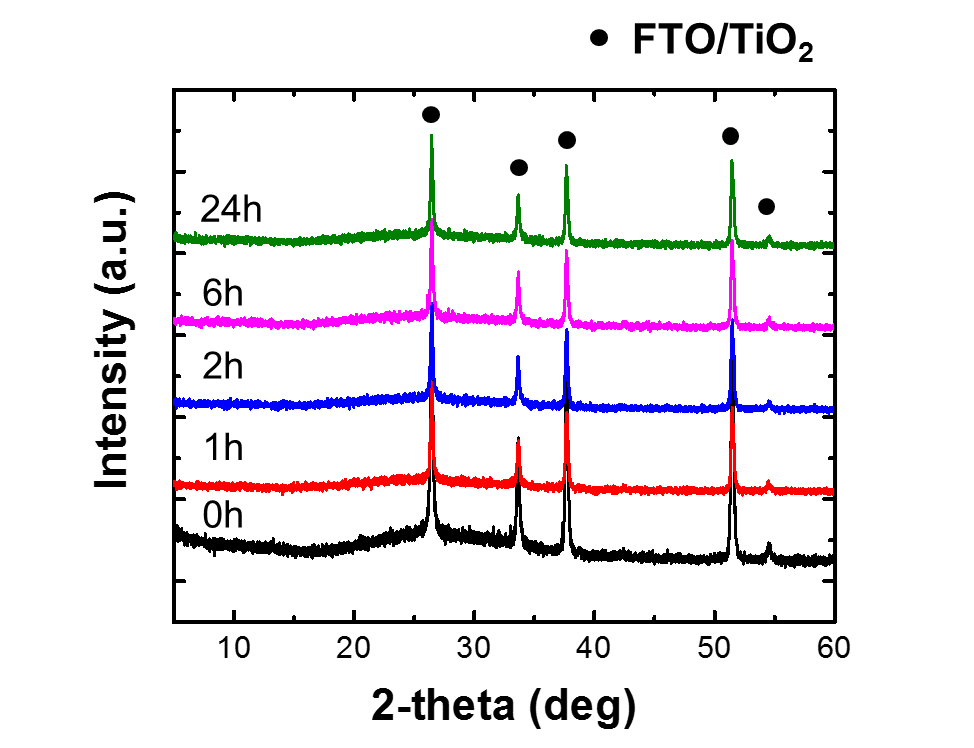


**Figure S6.** X-ray diffraction patterns of the FTO/TiO_2_/spiro-OMeTAD structure at 85 °C over time.


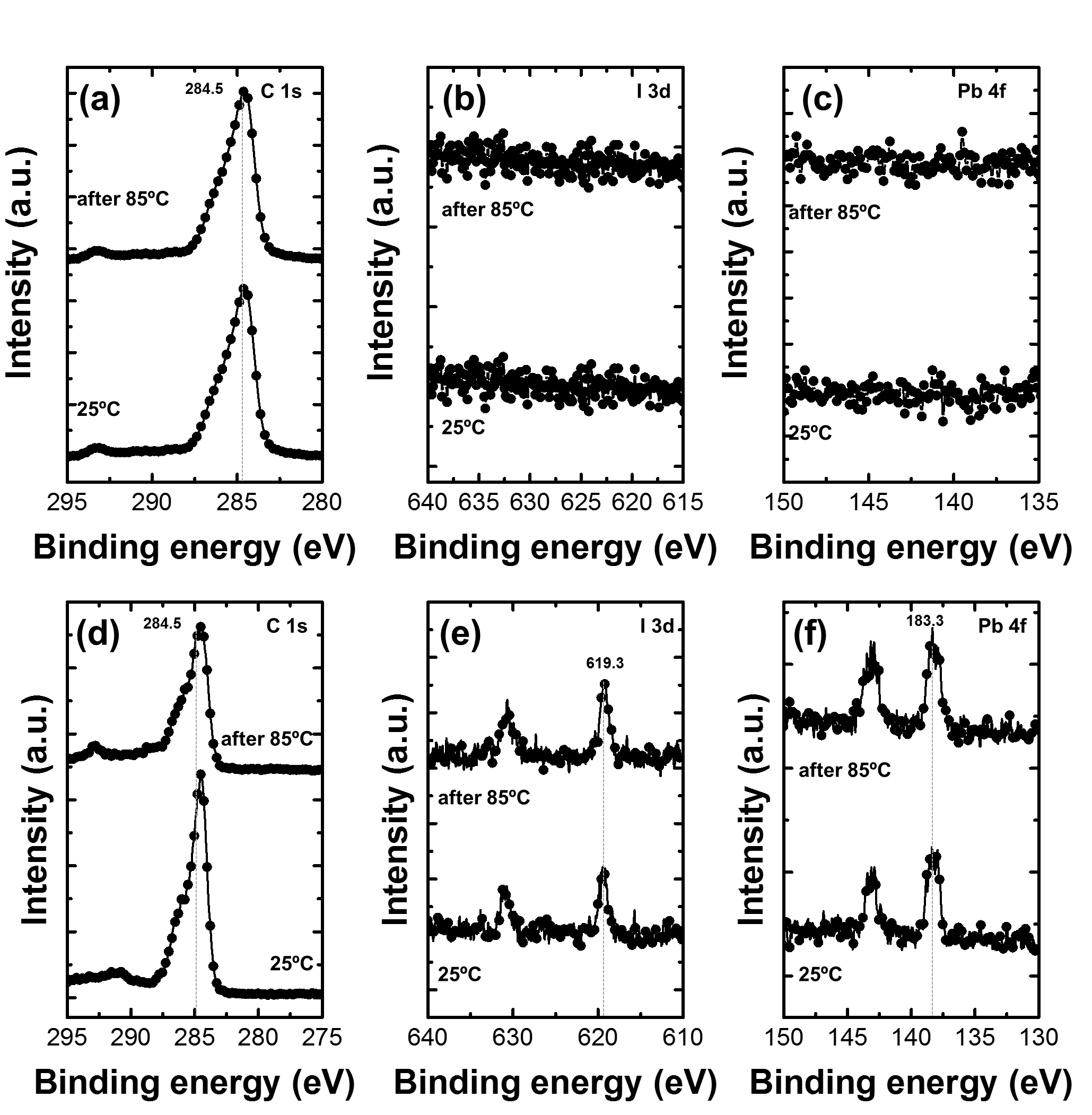


**Figure S7.** XPS narrow scan spectra of the as-prepared compound (25 °C) and after thermal treatment (85 °C): (a) C 1s, (b) I 3d, and (c) Pb 4f of spiro-OMeTAD only and (d) C 1s, (e) I 3d, and (f) Pb 4f of MAPbI_3_/spiro-OMeTAD. All XPS data were acquired from the spiro-OMeTAD side.


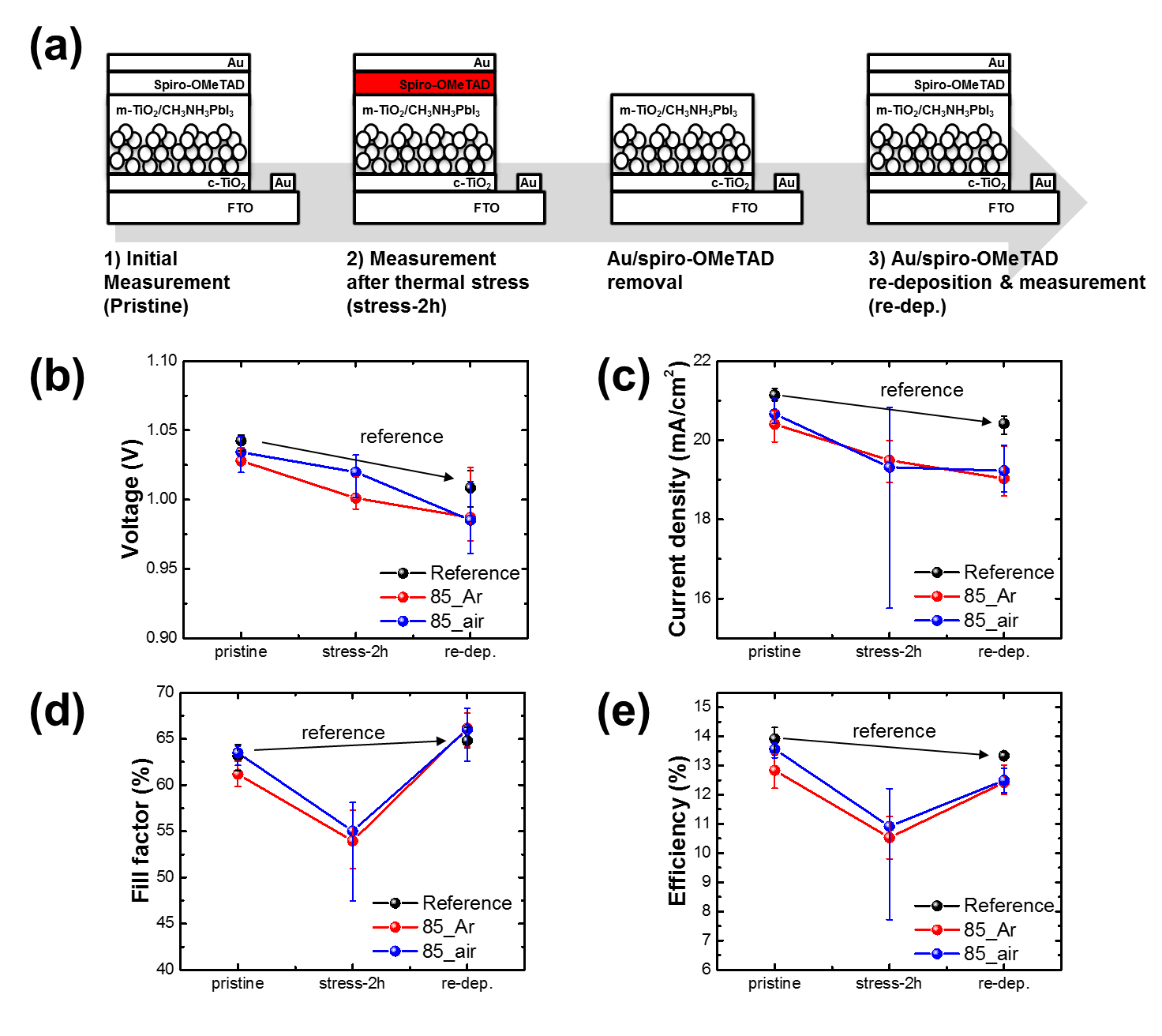


**Figure S8.** (a) Step-by-step procedure for the re-deposition of thermally degraded spiro-OMeTAD. 1) *I–V* curve for the pristine compound and 2) after 85 °C for 2 h under inert Ar and ambient air conditions. For fresh spiro-OMeTAD, Au and spiro-OMeTAD were removed using scotch tape and chlorobenzene. 3) *I–V* curve after re-deposition of spiro-OMeTAD and Au. According to the step-by-step procedures, *I–V* was plotted as (b) *V_oc_*, (c) *J_sc_*, (d) *FF*, and (e) efficiency.


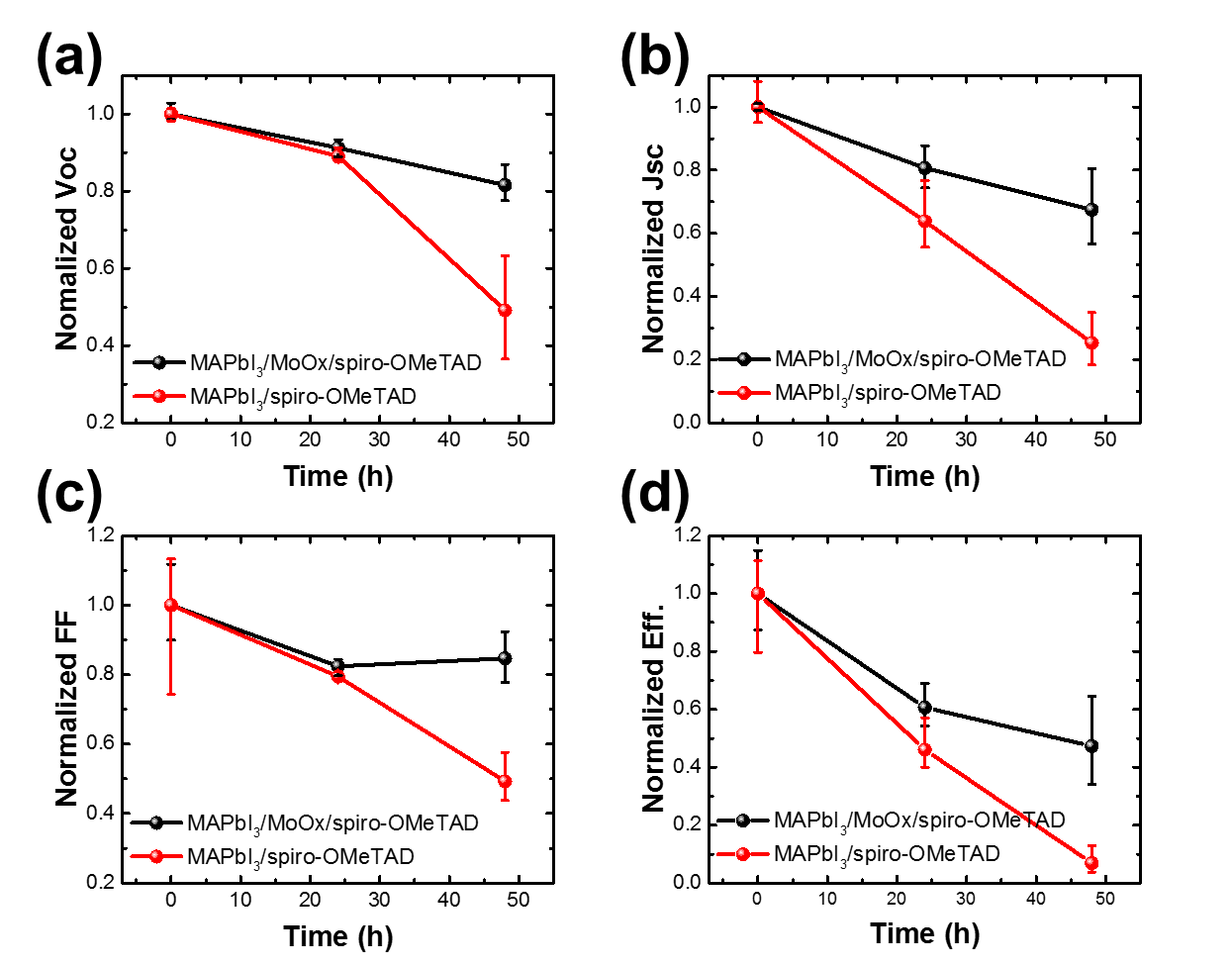


**Figure S9.** Long-term stability of TiO_2_/MAPbI_3_/spiro-OMeTAD– and TiO_2_/MAPbI_3_/MoO_x_/spiro-OMeTAD–structured PSCs: (a) normalized *V_oc_*, (b) normalized *J_sc_*, (c) normalized *FF*, and (d) normalized efficiency at 85 °C over time.


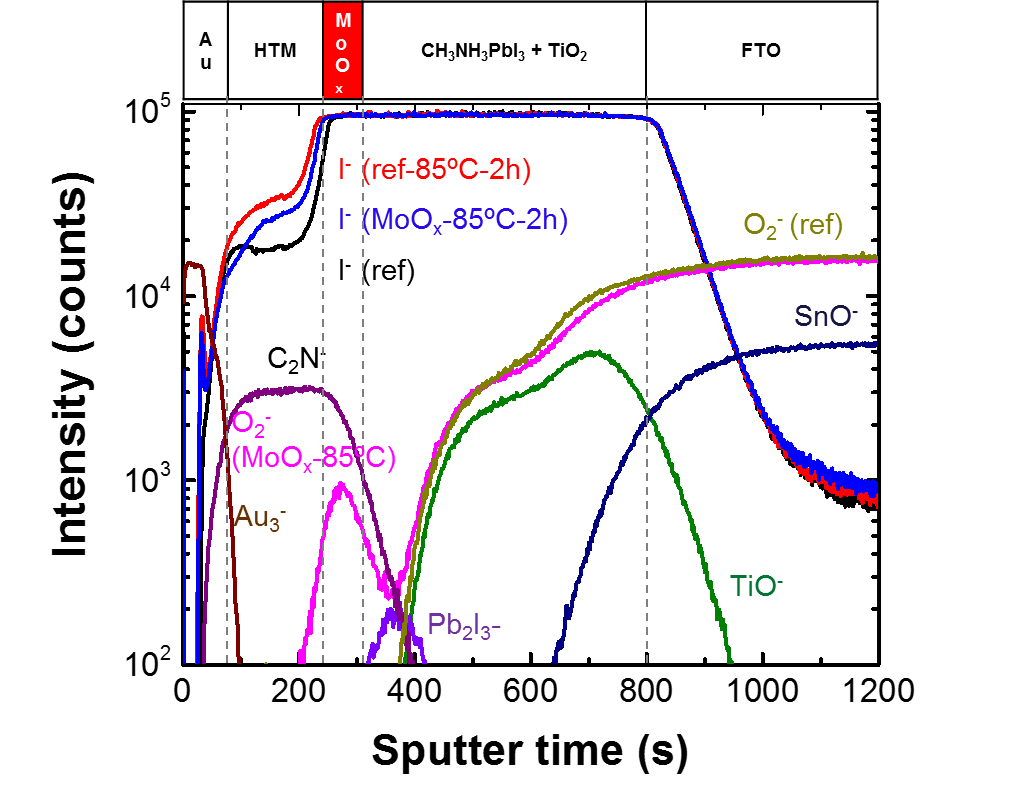


**Figure S10.** TOF-SIMS depth profiles of the concentrations of selected species for the reference cell (ref; without MoO_x_), thermally treated (85 °C) reference cell (ref-85°C-2h; without MoO_x_) and MoO_x_ cell (MoO_x_-85°C-2h; with MoO_x_).

Table S1. Average J-V characteristics of TiO_2_/MAPbI_3_/MoOx/spiro-OMeTAD-structured PSCs at 85 °C with aging time.

| **Aging time (h)** | **V_oc_ (mV)** | **J_sc_ (mA/cm^2^)** | **FF (%)** | **Eff. (%)** |
| --- | --- | --- | --- | --- |
| **0** | 1012 | 17.32 | 62.49 | 10.97 |
| **24** | 923.2 | 13.97 | 51.49 | 6.655 |
| **48** | 825.4 | 11.69 | 52.90 | 5.190 |
